# Using stacked deep learning models based on PET/CT images and clinical data to predict EGFR mutations in lung cancer

**DOI:** 10.3389/fmed.2022.1041034

**Published:** 2022-10-10

**Authors:** Song Chen, Xiangjun Han, Guangwei Tian, Yu Cao, Xuting Zheng, Xuena Li, Yaming Li

**Affiliations:** ^1^Department of Nuclear Medicine, The First Hospital of China Medical University, Shenyang, China; ^2^Department of Interventional Radiology, The First Hospital of China Medical University, Shenyang, China; ^3^Department of Radiation Oncology, The First Hospital of China Medical University, Shenyang, China; ^4^School of Information and Control Engineering, Liaoning Petrochemical University, Fushun, China; ^5^Department of Infectious Disease, The First Hospital of China Medical University, Shenyang, China

**Keywords:** positron-emission tomography, *EGFR* mutation, stack generalization, deep learning model, lung cancer

## Abstract

**Purpose:**

To determine whether stacked deep learning models based on PET/CT images and clinical data can help to predict epidermal growth factor receptor (EGFR) mutations in lung cancer.

**Methods:**

We analyzed data from two public datasets of patients who underwent ^18^F-FDG PET/CT. Three PET deep learning ResNet models and one CT deep learning ResNet model were trained as low-level predictors based on PET and CT images, respectively. A high-level Support Vector Machine model (Stack PET/CT and Clinical model) was trained using the prediction results of the low-level predictors and clinical data. The clinical data included sex, age, smoking history, SUVmax and SUVmean of the lesion. Fivefold cross-validation was used in this study to validate the prediction performance of the models. The predictive performance of the models was evaluated by receiver operator characteristic (ROC) curves. The area under the curve (AUC) was calculated.

**Results:**

One hundred forty-seven patients were included in this study. Among them, 37/147 cases were EGFR mutations, and 110/147 cases were EGFR wild-type. The ROC analysis showed that the Stack PET/CT & Clinical model had the best performance (AUC = 0.85 ± 0.09), with 0.76, 0.85 and 0.83 in sensitivity, specificity and accuracy, respectively. Three ResNet PET models had relatively higher AUCs (0.82 ± 0.07, 0.80 ± 0.08 and 0.79 ± 0.07) and outperformed the CT model (AUC = 0.58 ± 0.12).

**Conclusion:**

Using stack generalization, the deep learning model was able to efficiently combine the anatomic and biological imaging information gathered from PET/CT images with clinical data. This stacked deep learning model showed a strong ability to predict *EGFR* mutations with high accuracy.

## Introduction

Lung cancer is one of the most common malignancies worldwide and accounts for 18% of cancer-related deaths. More than 80% of lung cancer cases are non-small-cell lung cancer (NSCLC). In recent years, with the growing understanding of genetic changes, the management of lung cancer has made great advancements. Several genetic changes, including anaplastic lymphoma kinase (*ALK*) rearrangements and Kirsten rat sarcoma (*KRAS*) and epidermal growth factor receptor (*EGFR*) mutations, have been proven to be prognostic indicators for NSCLC patients. Targeted therapy using novel targeted drugs and immunotherapeutic agents has greatly improved the prognosis of lung cancer patients and has become an important aspect of personalized treatment.

Prior to targeted therapy, critical pathogenic gene mutation tests are suggested in patients with advanced NSCLC to guide the treatment. In NSCLC, the genes like *EGFR* and *KRAS* mutations, are important. Testing these important genes depends on routine biopsies or cytological examinations of tumors, which carry a few limitations. The biopsies and cytological examination are invasive tests. They are not always feasible and are often associated with a high risk of bleeding, pneumothorax and the collection of inadequate samples. Therefore, for patients who have recurrence after first-line treatment, rebiopsy of the recurrent lesion is not required, and targeted therapies might be executed based on the gene mutation test of the surgical specimen, assuming that no genetic changes occur between secondary tumors and recurrence. In these circumstances, the need to find a low-risk and non-invasive method to assess actionable biomarkers as a substitute for biopsies and cytological examination in NSCLC is emerging.

In recent years, researchers have utilized CT images to predict gene mutations, primarily by radiomic features and machine learning approaches. A systematic review showed that studies that used radiomic features of CT images to predict *EGFR* mutations had successfully obtained relatively accurate results (1). Scientists have also tried to use PET/CT images to predict gene mutations. Liu et al. employed random forest and logistic regression to predict *EGFR* mutations and found that predictive models based on radiomic features extracted from ^18^F-FDG PET/CT images achieved a satisfying prediction power in the identification of *EGFR* mutation status, with area under the curves (AUCs) ranging from 0.77 to 0.92 (2). Although radiomics and machine learning methods have successfully predicted some genetic mutations, the procedures to calculate the radiomic features and build the machine learning models are complicated and strict. These procedures are time-consuming and require full cooperation from both practiced imaging physicians and AI technicians, from detection, segmentation and feature extraction to feature selection and model optimization. Furthermore, radiomic features are sensitive to reconstruction parameters and have low repeatability between different PET/CT scanners (3). Thus, the machine learning models built on the radiomic features showed low receptibilities between different PET/CT centers.

Deep learning has achieved significant achievement in the AI industry in recent years as a result of its excellent images analysis capabilities, which allow researchers to bypass the arduous procedures of features calculation and features selection. In the field of imaging prediction of gene mutations, deep learning approaches have been continuously developed. In lung cancer, using CT images to train deep learning models and predict *EGFR* mutations has been studied and shows promising performance (4, 5).

In this study, we developed a deep learning-based model to predict *EGFR* mutation status in patients with lung cancer using ^18^F-FDG PET/CT scans and multiple public datasets. We evaluated the performance of the deep learning-based *EGFR* prediction model, which integrated the information extracted from PET/CT images and clinical data. We hope that this deep learning-based model will aid doctors in identifying suitable advanced lung cancer patients for *EGFR*-targeted therapy, allowing for more efficient and convenient application of precise medicine.

## Materials and methods

### Data collection

This study used two publicly available datasets selected from the Cancer Imaging Archive (TCIA). PET/CT data. The “TCGA-LUAD” dataset (6) and “NSCLC Radiogenomics” dataset (7) were used in this study, from which we obtained a collection of patients with clinical data and PET/CT images. The PET/CT images were acquired with 3 different PET/CT scanners. For every lesion, a semiautomatic segmentation method was used to delineate the lesion (8). Then, the delineations were reviewed by a board-certified nuclear medicine physician (with more than 10 years of working experience in nuclear medicine).

### Prepare dataset for training, validation and testing

Nested five-fold cross-validation was used to train and evaluate the prediction ability of the models. A five-folds cross validation were used as the inner loop, and another five-folds cross validation were used as the outer loop. All the lesions in this study were randomly separated into inner loop dataset and outer loop testing dataset using five-folds cross validation as the outer loop. And then the lesions in inner loop dataset were divided into training dataset (80%) and inner loop testing dataset (20%) with another five-folds cross validation as inner loop. The models were trained and optimized using the inner loop and tested using outer loop test dataset. In order to prevent over-fitting, 25% of lesions in training dataset of inner loop were randomly selected as validation dataset when training and optimizing the deep learning models. And the performances of the models were evaluated using outer loop test datasets.

### *EGFR* status prediction with PET/CT images and deep learning models

For each lesion we studied, we extracted three 2D slices from 3 planes (transverse, sagittal, and axial) at the largest slice of each lesion, which were used to characterize the imaging information of the lesion.

The images were normalized before being fed into the models using the following methods (9). The CT values in ROIs were converted into Hounsfield units, and the values ranging from −600 to 1,600 were transformed to [0, 1]; the PET values in the ROIs were converted into standard uptake values, and the values ranging from −0 to maximum value were transformed to [0, 1].

The deep learning models were trained using the images we extracted. The images of the lesion were first resampled to 32 × 32 pixels. Then, they were fed into the models. To reduce overfitting, data augmentation was applied in training dataset, and the images in the training dataset randomly proceeded with width/height-shift with 0~6 pixels, horizontal-flip, rotation with 0~20 degrees, and zoom with 100~120%.

Three PET Resnet models were built using PET images with Resnet 20 architecture, ResNet 32 architecture and ResNet 50 architecture. We also build a ResNet 32 CT model using CT images. Then, we used the stacked generalization method to build two SVM models. The first SVM model (stack PET/CT model) was trained using the prediction results of three PET models and one CT model together. The second SVM model (Stack PET/CT and clinical model) was trained by the prediction results of three PET models and one CT model and clinical data. The clinical data included age, smoking history, the sex of each patient, the SUVmax value and the SUVmean value of each lesion.

The ResNet models were trained with a batch size of 32. The Adam optimizer was used in this study to update the parameters of the deep learning models. The training was stopped after 50 epochs or the accuracy of the validation dataset prediction stop increasing for 10 epochs.

After trained and evaluated the performances of the ResNet models, stacked generalization was used to integrate the results of ResNet models to further improve the prediction ability. Stacked generalization is an ensemble method that allows researchers to combine several different prediction models (lower-level classifier) into one to achieve higher predictive accuracy (10). In this study, the results of ResNet PET models and ResNet CT model served as lower-level classifiers. The support vector machine (SVM) served as the higher-level classifier model to make a new prediction based on the prediction results of the lower-level classifiers.

### *EGFR* status prediction with radiomics features of PET/CT images and machine learning models

The radiomic features were extracted using Python (version 3.7). The PyRadiomics module was used to calculate the radiomic features. All the images were resampled to the same size before feature extraction. The PET images were resampled to 3.9063 × 3.9063 × 3.27 mm. The CT images were resampled to 0.98 × 0.98 × 3.27 mm. The bin sizes were 0.1 and 15 for PET and CT images, respectively. A total of 120 radiomic features were extracted from the PET and CT images for each lesion, respectively. These radiomic features include: first order statistics (19 features), shape-based (3D) (16 features), shape-based (2D) (10 features), gray level cooccurrence matrix (24 features), gray level run length matrix (16 features), gray level size zone matrix (16 features), neighboring gray tone difference matrix (5 features) and gray level dependence matrix (14 features).

For comparison, we trained machine learning model based on radiomic features for *EGFR* mutation prediction. The machine learning model was trained and tested using the same method with previous study (11). In order to reduce the size of the dataset, sequential forward floating selection (SFFS) was used to select only a few critical features to training the Support Vector Machine (SVM) models. The feature selection was performed in the training dataset (80% data) and testing dataset (20% data) of the inner loop, the SFFS stopped when the performance of the SVM decreased. Using those selected radiomic features, an SVM model was trained and then evaluated using the testing dataset of the outer loop.

### Software tools and statistical analysis

Models were trained and validated using Python (version 3.7). The scikit-learn module was used to train the SVM models based on clinical features and to integrate the results of the lower-level classifiers (ResNet models). The implementation of the deep learning models (ResNet models) was performed by the Keras toolkit.

Statistical analysis was performed by using Python (version 3.7) and SPSS (version 17.0). The scikit-learn package was used for ROC analysis. Meanwhile, the matplotlib package was employed to plot the ROC curve. The predictive performance of deep learning models and machine learning models was evaluated by receiver operator characteristic (ROC) curves. The area under the curve (AUC) was calculated. The optimal diagnosis threshold for each model was calculated using the Youden Index. By employing the optimal threshold, the sensitivity, specificity and accuracy were calculated according to the ROC curve. Chi-Square test was performed to compare the differences between two categorical variables. *T* test was performed to compare the differences between two normal distribution samples. And Mann-Whitney test was performed to compare the differences between two independent samples when the sample distributions are not normally distributed.

## Results

### Clinical characteristics of patients

**Figure 1:**
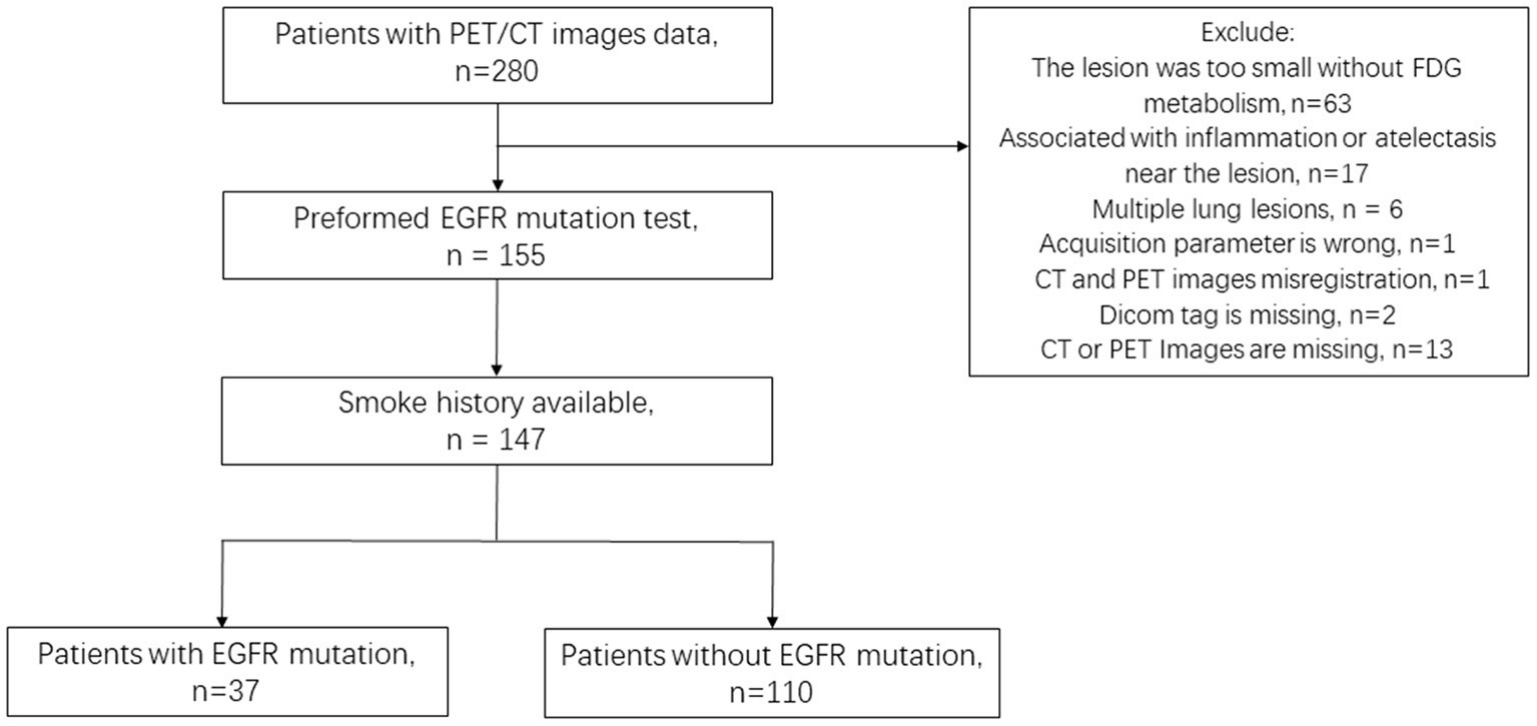
Flowchart of patient selection. EGFR, epidermal growth factor receptor.

PET/CT images and clinical data from the NSCLC Radiogenomic dataset and TCIA-LUAD dataset were reviewed, in which 147 patients (135 patients from NSCLC Radiogenomic dataset, 12 patients from TCIA-LUAD dataset) satisfied the experimental requirements: (1) PET/CT images and clinical information integrity; (2) Lesions were larger than 10 voxels on PET images; (3) The EFGR mutation was tested. Finally, 147 lesions from 147 patients (age: 68.57 ± 9.78) were included in this study. A total of 37/147 had *EGFR* mutations, and 110/147 were wild-type *EGFR* (Figure 1).

According to the *EGFR* mutation status the patients were divided into *EGFR*-mutation group and EFGR wild-type group (Table 1). Mann-Whitney test showed that the SUVmax value of *EGFR* mutant group is significantly higher that *EGFR* wild-type group (Z = −0.24, *P* = 0.016). There was no statistical difference of SUVmean value between two groups (Z = −0.37, *P* = 0.714). T tested showed that there was no statistically difference in age between two groups (T = 0.02, *P* = 0.987). Chi-Square test showed that sex and smoke status had a significantly difference between two groups (X = 6.948, *P* = 0.008; X = 29.30, *P* = 0.000), the *EGFR* mutation group had more female patients and more non-smokers.

**Table 1:** Clinical characteristics of patients.

	EGFR^*^-mutation group	EGFR^*^wild-type group	***p*-Value**
Number of cases	37	110	
Sex			0.008
Male	17	77	
Female	20	33	
Age	68.59 ± 9.33	68.56 ± 9.97	0.987
SUVmax	5.87 ± 5.46	8.28 ± 6.77	0.016
SUVmean	0.86 ± 0.40	0.90 ± 0.46	0.714
Smoke status			0.000
Non-smoker	20	13	
Former	15	73	
Current	2	24	

* EGFR, epidermal growth factor receptor.

### Characteristics of images

The reconstruction information of the image data was extracted from the dicom files.

For the resolution of PET images, 54 cases were reconstructed into 3.65 × 3.65 × 3.27 mm; 37 cases were 5.47 × 5.47 × 3.27 mm; 23 cases were 3.90 × 3.90 × 3.27 mm; and 33 cases were reconstructed into other resolutions (Table 2). For the resolution of CT images, 112 cases were reconstructed into 0.98 × 0.98 × 3.27 mm, and 35 cases were reconstructed into other resolutions (Table 2).

**Table 2:** Characteristics of the images.

	EFGR^*^Wildtype	EGFR^*^Mutation	**Total**
**Resolution of PET images**			
3.65 × 3.65 × 3.27 mm	35	19	54
3.91 × 3.91 × 3.27 mm	19	4	23
3.91 × 3.91 × 4.25 mm	1	0	1
4.00 × 4.00 × 4.00 mm	2	2	4
4.06 × 4.06 × 3.27 mm	1	0	1
4.07 × 4.07 × 3 mm	3	1	4
4.07 × 4.07 × 4 mm	0	1	1
4.07 × 4.07 × 5 mm	1	2	3
4.3 × 4.3 × 4.25 mm	7	1	8
4.69 × 4.69 × 3.27 mm	2	0	2
5.15 × 5.15 × 3.38 mm	2	0	2
5.31 × 5.31 × 2.5 mm	1	1	2
5.31 × 5.31 × 3.4 mm	3	2	5
5.47 × 5.47 × 3.27 mm	32	5	37
**Matric size of PET image**			
128 × 128	68	13	81
144 × 144	2	2	4
168 × 168	4	4	8
192 × 192	35	19	54
**Resolution of CT images**			
0.75 × 0.75 × 3.27 mm	1	0	1
0.87 × 0.87 × 0.62 mm	1	0	1
0.88 × 0.88 × 5 mm	2	1	3
0.91 × 0.91 × 3.27 mm	0	1	1
0.98 × 0.98 × 2 mm	0	1	1
0.98 × 0.98 × 2.5 mm	3	1	4
0.98 × 0.98 × 3 mm	2	0	2
0.98 × 0.98 × 3.27 mm	86	26	112
0.98 × 0.98 × 3.4 mm	3	2	5
0.98 × 0.98 × 4 mm	1	3	4
0.98 × 0.98 × 4.25 mm	8	1	9
1.17 × 1.17 × 2.5 mm	0	1	1
1.37 × 1.37 × 3.27 mm	1	1	2
1.68 × 1.68 × 0.98 mm	1	0	1

* EGFR, epidermal growth factor receptor.

### The predictive performance of models

**Figure 2:**
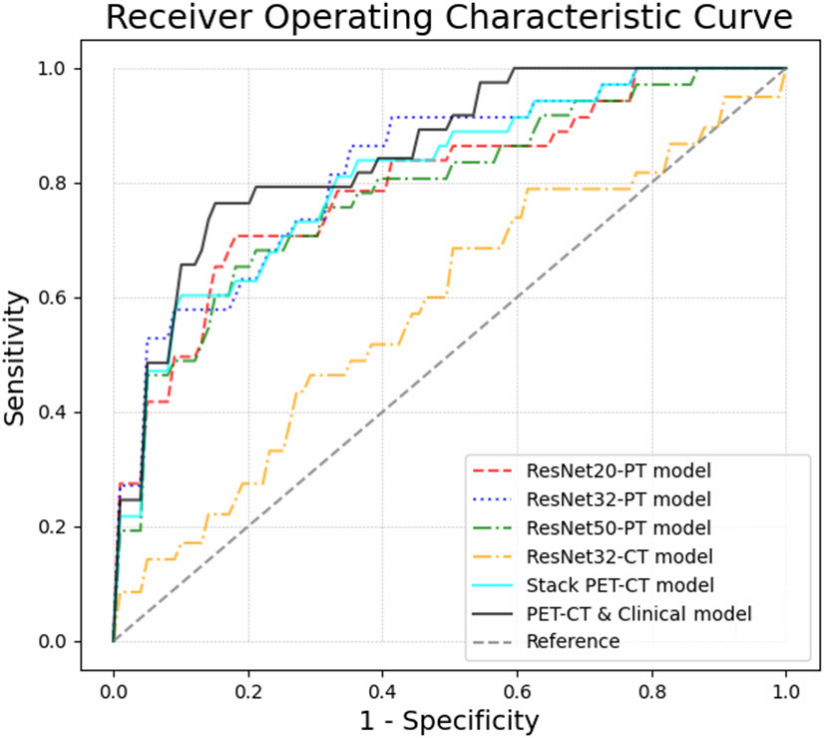
The ROC curves of different models in 5-fold cross validation. By integrating the clinical information and images, the Stack PET/CT and Clinical model obtained the highest AUC at 0.85 ± 0.09. The stacked PET/CT model and ResNet PET models also obtained relatively high AUCs of 0.79~0.82 ± 0.07~0.08. The ResNet CT models and radiomic model showed low predictive ability.

The performance of the models can be compared by sensitivity, specificity, accuracy and the area under the ROC curve (AUC) (Table 3 and Figure 2). The ROC analysis showed that the three Resnet PET models and the stack PET/CT model had higher AUCs and significantly outperformed the CT model and radiomic model. The AUC of Resnet 32 CT model is similar with radiomics model. After integrated clinical data (age, smoking history, and sex) into the stack PET/CT model we got the stack PET/CT and Clinical model. Compare to stack PET/CT model, the performance of the stack PET/CT and Clinical model improved, and the AUC increased from 0.81 to 0.85, the sensitivity and accuracy of the stack PET/CT and Clinical Data model increased from 0.60 to 0.76 and 0.82 to 0.83, respectively. The Stack PET/CT and Clinical model had the highest specificity and accuracy and a relatively high sensitivity.

**Table 3:** The performance of different models to classify epidermal growth factor receptor-mutated lesions.

**Models**	AUC^*^	**Number of ture/false positive cases**	**Number of ture/false negative cases**	**Sensitivity**	**Specificity**	**Accuracy**
Stack PET/CT and Clinical model	0.85 ± 0.09	28/9	94/16	0.76	0.85	0.83
Stack PET/CT model	0.81 ± 0.07	22/15	99/11	0.60	0.90	0.82
Resnet 20 PET model	0.80 ± 0.08	26/11	90/20	0.70	0.82	0.79
Resnet 32 PET model	0.82 ± 0.07	32/5	71/39	0.86	0.65	0.70
Resnet 50 PET model	0.79 ± 0.07	24/13	90/20	0.65	0.82	0.78
Resnet 32 CT model	0.58 ± 0.12	26/11	54/56	0.69	0.49	0.54
Radiomic model	0.60 ± 0.06	12/25	95/15	0.32	0.86	0.73

* AUC, The Area Under the Curve.

## Discussion

In this study, we proposed a prediction model based on ResNet deep learning models to predict *EGFR* mutation status using non-invasive ^18^F-FDG PET/CT images and clinical data of patients with lung cancer. By using stacked generalization, the prediction model integrated the medical images and clinical data together and showed encouraging results and strong performance in predicting *EGFR* mutations. To our knowledge this is the first study that predicted the *EGFR* status based on the stacked deep learning models which integrated the medical images and clinical data from multiple centers together.

For patients with advanced pulmonary adenocarcinoma, radiotherapy and chemotherapy remain the major treatment. Molecular-targeted medicines, such as *EGFR*-TKIs, have considerably improved the outcome of advanced lung cancer patients compared to standard therapy. The effectiveness of *EGFR*-TKIs is dependent on the presence of an *EGFR* mutation. Using image-based AI models to predict *EGFR* mutation could be relevant when invasive examinations are contraindicated.

The deep learning model has been demonstrated to be a useful tool to analyze medical images. The performance of the deep learning models was affected by the quality of the dataset. Usually, training a good deep learning model requires a large image dataset with good quality control. A systemic review (1) showed that until 2020, four studies used CT images to build deep learning models to predict *EGFR*, but no one had used PET/CT images to train deep learning models. The reason for this might be it is hard to integrate PET and CT images together to train a deep learning model. In order to do that we used stacked generalization in this study. Stacked generalization was designed to combine several different prediction models into one. Although the concept of “stacked generalization” was originally developed in 1992, the theoretical guarantees for stacking were not proven until the publication of a paper titled, “Super Learner,” in 2007. Stacked generalization was then proven to be an effective approach to improve the predictive performance (10, 12). The stacked generalization is to integrate the results of several “lower-level” predictive models into a “higher-level” model. The goal in stacking is to ensemble strong, diverse sets of “lower-level” predictive models together. The benefit of stacked generalization is that it can harness the capabilities of a range of well-performing models on a classification or regression task and make predictions that have better performance than any single model. Traditional stacking is used to integrate the results of several models trained by the same dataset. However, in this study, we used stack generalization to integrate the data from different datasets (images and clinical information). These data reflect different features of the lesions. PET images reflect biological information, CT images reflect anatomic information, and clinical data reflect possible predisposing factors. In this study, stacked generalization helped the models to integrate anatomic, biological imaging data and clinical information. This is a successful attempt. After integrating the information of images and clinical data, the AUC of the model was improved, proving that integrating multiple aspect information of lesions could further improve the predicting performance.

*EGFR* mutation prevalence had associated with gender and smoking status. The prevalence was higher in females (females vs. males: 43.7 vs. 24.0%) and non-smokers (non-smoker vs. past or current smoker: 49.3 vs. 21.5%) (13). In this study we found that clinical data (age, smoking history, and sex) helped the PET/CT and Clinical model to get a better predictive performance. After integrated clinical data into the stack PET/CT model, the AUC increased from 0.81 to 0.85, and the sensitivity and accuracy increased from 0.60 to 0.76 and 0.82 to 0.83, respectively.

Using PET/CT radiomics features to predict *EGFR* mutations showed a promised result with an AUC ranging from 0.83 to 0.79 (1, 14–16). But radiomic features are highly sensitive to the reconstruction method, which means those radiomic models might not be reliable when used on different PET/CT scanners with different reconstruction parameters (3). In this study, the imaging data came from several different hospitals. The PET reconstruction parameter was different between different hospitals. This might be the reason that the radiomic model showed low predictive ability in this study. And compare to radiomics model, the deep learning models showed a better performance to deal with images with difference reconstruction parameters.

Previous studies have shown that using deep learning model trained by CT images to predict *EGFR* mutations had a promising result, with AUC ranging from 0.85 to 0.75 (1, 4). But in this study, the CT images showed a very weak ability to predict *EGFR* mutations. The reason for this might be that the quality and quantity of the CT images of PET/CT are not sufficient for training the deep learning models. The CT images of PET/CT is free breath CT image. And some of the CT images in this study are contrast-enhanced CT, and some of them are not. This might be another reason for the poor performance of the ResNet CT model.

In this study we used nested cross validation to train and build our models. Compare non-nested cross validation, model selection without nested cross validation uses the same data to tune model parameters and evaluate model performance. Information of test dataset may thus “leak” into the model and overfit the data. The magnitude of this effect is primarily dependent on the size of the dataset and the stability of the model. To avoid this problem, nested cross validation effectively uses a series of train/validation/test set splits. In the inner loop, the models were trained and optimized. In the outer loop, performance of the model is estimated by averaging test set scores over several dataset splits. This is a useful method to train and test the models when the dataset is small. But compared to data split method, nested cross validation consumed more time in training and testing the models.

In this study is that the segmentation method of this study is semiautomatic. This semiautomatic approach requires that the physician give an approximate location of lesions. Li et al. reported that a deep learning model can be trained to segment tumors on PET/CT images (17). Instead of the semiautomatic segmentation approach, we should build another deep learning model to segment the lesion in future research. By integrate several deep learning models, an automatic non-invasive decision support system could be developed, including identification, segmentation and *EGFR* status prediction. Using stack generalization to integrate more models based on multimodality imaging, radiomic features and more clinical features together may further improve the predictive performance.

## Conclusion

In this study, we trained a deep learning-based algorithm to predict the *EGFR* mutation status in patients with lung cancer using ^18^F-FDG PET/CT images from multiple hospitals. Using stack generalization, the machine learning model was able to efficiently combine the anatomic and biological imaging information gathered from CT and PET images with clinical data. This model showed the ability to assist doctors in predicting *EGFR* mutations non-invasively to identify patients with lung cancer who would benefit from *EGFR*-TKI therapy.

## Data availability statement

The original contributions presented in the study are included in the article/supplementary material, further inquiries can be directed to the corresponding author.

## Author contributions

SC, YC, XL, and YL contributed to the study conception and design. SC, XL, XH, and GT carried out the data collection and imaging segmentation. SC and YC carried out the stacked generalization, training, and testing of models. XZ performed the statistical analysis and figure editing. The first draft of the manuscript was written by SC. All authors commented on previous versions of the manuscript, read, and approved the final manuscript.

## Funding

This work was supported by the National Natural Science Foundation of China (Nos. 81901816 and 81901846) and Natural Science Foundation of Liaoning Province of China (2019-MS-383).

## Conflict of interest

The authors declare that the research was conducted in the absence of any commercial or financial relationships that could be construed as a potential conflict of interest.

## Publisher's note

All claims expressed in this article are solely those of the authors and do not necessarily represent those of their affiliated organizations, or those of the publisher, the editors and the reviewers. Any product that may be evaluated in this article, or claim that may be made by its manufacturer, is not guaranteed or endorsed by the publisher.

## Abbreviations

ALK: Anaplastic Lymphoma Kinase
EGFR: Epidermal Growth Factor Receptor
FDG: Flourodeoxyglucose
KRAS: Kirsten Rat Sarcoma
ResNet: Residual Networks
ROC: Receiver Operation Curve
VOI: Volume Of Interest.

## References

[1] Ninatti G Kirienko M Neri E Sollini M Chiti A. Imaging-based prediction of molecular therapy targets in NSCLC by radiogenomics and AI approaches: a systematic review. Diagnostics. (2020) 10:359. 10.3390/diagnostics10060359 32486314 PMC7345054

[2] Liu Q Sun D Li N Kim J Feng D Huang G. Predicting EGFR mutation subtypes in lung adenocarcinoma using (18)F-FDG PET/CT radiomic features. Transl Lung Cancer Res. (2020) 9:549–62. 10.21037/tlcr.2020.04.17 32676319 PMC7354146

[3] Lin C Bradshaw T Perk T Harmon S Eickhoff J Jallow N. Repeatability of quantitative 18F-NaF PET: a multicenter study. J Nucl Med. (2016) 57:1872–9. 10.2967/jnumed.116.177295 27445292 PMC6952054

[4] Dong Y Hou L Yang W Han J Wang J Qiang Y. Multi-channel multi-task deep learning for predicting EGFR and KRAS mutations of non-small cell lung cancer on CT images. Quant Imaging Med Surg. (2021) 11:2354–75. 10.21037/qims-20-600 34079707 PMC8107307

[5] Zhao W Yang J Ni B Bi D Sun Y Xu M. Toward automatic prediction of EGFR mutation status in pulmonary adenocarcinoma with 3D deep learning. Cancer Med. (2019) 8:3532–43. 10.1002/cam4.2233 31074592 PMC6601587

[6] Albertina B Watson M Holback C Jarosz R Kirk S Lee Y. Radiology data from the cancer genome atlas lung adenocarcinoma [TCGA-LUAD] collection. In: Archive TCI (2016).

[7] Bakr SG Olivier Echegaray Sebastian Ayers Kelsey. Data for NSCLC radiogenomics collection. In: Archive TCI (2017).

[8] Beichel RR Van Tol M Ulrich EJ Bauer C Chang T Plichta KA. Semiautomated segmentation of head and neck cancers in 18F-FDG PET scans: a just-enough-interaction approach. Med Phys. (2016) 43:2948–64. 10.1118/1.4948679 27277044 PMC4874930

[9] Yin G Wang Z Song Y Li X Chen Y Zhu L. Prediction of EGFR mutation status based on (18)F-FDG PET/CT imaging using deep learning-based model in lung adenocarcinoma. Front Oncol. (2021) 11:709137. 10.3389/fonc.2021.709137 34367993 PMC8340023

[10] Naimi AI Balzer LB. Stacked generalization: an introduction to super learning. Eur J Epidemiol. (2018) 33:459–64. 10.1007/s10654-018-0390-z 29637384 PMC6089257

[11] Chen S Harmon S Perk T Li X Chen M Li Y. Diagnostic classification of solitary pulmonary nodules using dual time 18F-FDG PET/CT image texture features in granuloma-endemic regions. Sci Rep. (2017) 7:9370. 10.1038/s41598-017-08764-7 28839156 PMC5571049

[12] van der Laan MJ Polley EC Hubbard AE. Super learner. Stat Appl Genet Mol Biol. (2007). 10.2202/1544-6115.1309 17910531

[13] Zhang YL Yuan JQ Wang KF Fu XH Han XR Threapleton D. The prevalence of EGFR mutation in patients with non-small cell lung cancer: a systematic review and meta-analysis. Oncotarget. (2016) 7:78985–93. 10.18632/oncotarget.12587 27738317 PMC5346692

[14] Yip SSF Parmar C Kim J Huynh E Mak RH Aerts H. Impact of experimental design on PET radiomics in predicting somatic mutation status. Eur J Radiol. (2017) 97:8–15. 10.1016/j.ejrad.2017.10.009 29153372

[15] Jiang M Zhang Y Xu J Ji M Guo Y Guo Y. Assessing EGFR gene mutation status in non-small cell lung cancer with imaging features from PET/CT. Nucl Med Commun. (2019) 40:842–9. 10.1097/MNM.0000000000001043 31290849

[16] Mu W Jiang L Zhang J Shi Y Gray JE Tunali I. Non-invasive decision support for NSCLC treatment using PET/CT radiomics. Nat Commun. (2020) 11:5228. 10.1038/s41467-020-19116-x 33067442 PMC7567795

[17] Li L Zhao X Lu W Tan S. Deep learning for variational multimodality tumor segmentation in PET/CT. Neurocomputing. (2020) 392:277–95. 10.1016/j.neucom.2018.10.099 32773965 PMC7405839

